# Enhancing anatomy education with virtual reality: integrating three-dimensional models for improved learning efficiency and student satisfaction

**DOI:** 10.3389/fmed.2025.1555053

**Published:** 2025-06-04

**Authors:** Shuliang Niu, Jinlong Zhang, Jiang Lin, Binbin Wang, Jie Yan

**Affiliations:** ^1^Department of Forensic Science, School of Basic Medical Science, Central South University, Changsha, Hunan, China; ^2^School of Basic Medical Science, Xinjiang Medical University, Urumqi, China; ^3^School of Basic Medical Science, Xinjiang Second Medical College, Karamay, China

**Keywords:** digital technology, virtual reality, three-dimensional anatomy model, teaching methodology, anatomy education

## Abstract

Traditional anatomy education, which primarily relies on two-dimensional imagery, often struggles to effectively convey the complex spatial relationships of human anatomy. Virtual reality and three-dimensional (3D) anatomy models present a promising solution to these limitations. This study investigates the impact of integrating 3D anatomy models into a blended learning framework across pre-class, in-class, and post-class phases. A total of 169 medical students from Xinjiang Medical University were divided into three groups: a control group (Class A, *n* = 57) following a traditional blended learning approach, and two experimental groups: Class B (*n* = 56), which incorporated continuous 3D model integration, and Class C (*n* = 56), which adopted a phased 3D model integration strategy. Learning outcomes and student satisfaction were assessed through formative evaluations, surveys, and statistical analyses. Our analytical framework employed dual statistical validation protocols: parametric testing via independent samples t-tests for normally distributed data and non-parametric verification through Mann-Whitney U tests for skewed distributions. Class B achieved higher scores than Class A across two assessment stages (*p* < 0.05). In pre-class evaluations, Class B (*n* = 56) scored 69.7 ± 7.5 compared to Class A’s 63.8 ± 6.9 (*n* = 57). This performance gap persisted during in-class assessments, with Class B attaining 77.1 (± 8.7) against Class A’s 70.8 (± 7.6). Prior to the intervention, Class C (*n* = 56) exhibited a mean score of 61.8 ± 6.1, which increased to 67.0 ± 6.7 post-intervention. The score gaps demonstrate the teaching method’s effectiveness Class C demonstrated a statistically significant enhancement in pre-class assessment performance (*p* < 0.05) following the implementation of 3D anatomical modeling. However, no significant differences were observed among the groups in midterm or final exam scores (*p* > 0.05). Satisfaction scores in Class B were significantly higher than in Class A (*p* < 0.05), particularly in aspects of learning interest and teaching diversity. Class C also reported increased satisfaction in some dimensions after 3D model integration (*p* < 0.05). All survey instruments demonstrated good reliability (Cronbach’s alpha > 0.7). In conclusion, while 3D anatomy models enhance student engagement, learning efficiency, and overall satisfaction, their effect on long-term retention and final exam performance remains limited. These findings underscore the need for a strategic approach to integrating 3D technologies in anatomy education to maximize their educational benefits.

## 1 Introduction

The rapid development of digital technologies, fueled by Industry 4.0, is profoundly reshaping societal structures and transforming the workforce (1, 2). In education, the integration of these technologies holds transformative potential, offering new ways to enhance educational practices, reshape pedagogical relationships, and refine management strategies (3). These shifts align with constructivist learning theory (4), which highlights how digital tools can play a pivotal role in redefining educational paradigms and interactions (5). The COVID-19 pandemic accelerated the transition to online education, further promoting the digital transformation of teaching and learning (6–11). Among digital technologies, virtual reality (VR) technology has made rapid progress. VR creates computer-generated 3D environments that can be non-immersive, semi-immersive, or fully immersive, allowing users to explore realistic or fictional spaces. Desktop VR is one of the most basic forms of non-immersive VR, which lets users explore virtual worlds through a regular screen. For example, common 3D visualization models, such as those used in architectural design or medical training, fall under this category (12, 13).

Anatomy education, traditionally reliant on two-dimensional images and textual descriptions, faces challenges in helping students visualize and understand spatial relationships between structures (14, 15). The use of digital technologies, such as three-dimensional anatomy model, offers significant advantages in overcoming these challenges (1). 3D anatomy models, which present organs and tissues in stereoscopic views, have been found to improve comprehension and engagement compared to traditional methods (16, 17).

Numerous studies have explored the efficacy of 3D anatomy models in educational settings. Research indicates that 3D models can significantly support anatomy education, offering more effective alternatives to traditional learning tools (18–23). For instance, Agarwal et al. demonstrated that 3D-printed models improved learning outcomes in orthopedic education (24). Similarly, immersive virtual reality has been successfully applied to anatomy teaching (25).

Constructivism posits that learning is an active process of cognitive construction facilitated through environmental interactions (26). Blended learning, which combines face-to-face instruction with digital resources such as MOOCs and micro-lectures, operationalizes constructivist principles by emphasizing self-directed inquiry and multimodal interactions (27, 28). Compared to traditional classroom-only approaches, blended learning has demonstrated superior educational outcomes and is widely recognized as a valuable enhancement to conventional pedagogy (28). Its successful application across disciplines, particularly in medical education (29), underscores its adaptability and efficacy. However, a systematically designed blended learning framework incorporating 3D models to enhance pedagogical effectiveness remains underexplored (30).

Building upon a constructivist-based blended learning framework, this study integrated 3D anatomy models as a component of the pedagogical approach for anatomy education. This integration aligned with constructivist principles by fostering active engagement and learner-driven exploration of anatomical structures. To evaluate the intervention’s effectiveness, we conducted statistical analyses of formative assessments and survey questionnaires, adhering to post-positivist principles (31) through prioritizing statistically significant results as reliable evidence.

We aimed to achieve two primary research objectives: first, to assess the impact of 3D anatomy models on student engagement, learning efficiency, and retention in anatomy education; second, to develop a comprehensive blended learning framework that integrates 3D anatomy models into pre-class, in-class, and post-class activities to enhance the learning experience. To address these objectives, the study formulated the following research questions: (1) How does the integration of 3D anatomy models affect student engagement and learning efficiency in anatomy education? (2) What are the key components of an effective blended learning framework that incorporates 3D technology in anatomy education? (3) To what extent does the blended learning framework incorporating 3D technology enhance educational outcomes in anatomy education compared to traditional methods?

## 2 Methods

### 2.1 Population and sampling

This study examined three cohorts from the clinical medicine program at Xinjiang Medical University, which included Class A, Class B, and Class C, with a total of 169 participants. All three cohorts had no prior experience in anatomy education before the start of the study. The cohorts began their studies at the same time, exhibiting similar entrance scores, gender ratios, and other general demographic characteristics, with no significant statistical differences between the groups. The anatomy course was a compulsory module for all clinical medicine students and was administered by the Department of Anatomy at Xinjiang Medical University.

Class A (*n* = 57) served as the control group, following the traditional blended learning framework, which combined traditional two-dimensional illustrations and physical specimens with digital components such as MOOCs and micro-learning modules. Class B (*n* = 56) was experimental group one, using the traditional blended learning model enhanced by the continuous integration of 3D anatomy models throughout the course. Class C (*n* = 56) was experimental group two, which initially followed the traditional blended learning model for teaching the musculoskeletal, visceral, and endocrine systems (phase 1). Subsequently, the group transitioned to a blended learning model that incorporates 3D anatomy models for studying the vascular system, sensory organs, and nervous system (phase 2).

All three cohorts utilized the same blended learning resources, and all students were taught by the same group of faculty members. The primary variable distinguishing the groups was the application of the 3D anatomy models, which were integrated to varying extents across the groups. This design allowed for a comparison of the effectiveness of different 3D anatomy model integration strategies within a blended learning framework.

### 2.2 Preparation of teaching software

Instructors and students accessed anatomy education tools by searching “anatomy” in iOS/Google Play stores, with representative apps including Visible Body 3D Anatomy (Visbody, China) and Anatomy-3D Atlas (Catfish Animation Studio, Italy).

Pre-class and in-class assessments were conducted via “Rain Classroom,” an intelligent teaching platform (jointly developed by Tsinghua University and XuetangX) integrated with WeChat. This platform supported multimodal teaching tasks: distributing materials (PPT, videos, literature), managing assignments/assessments, hosting seminars, and conducting auto-analyzed quizzes (32). Additionally, “MOOC” was implemented to deliver structured learning objectives and resource repositories (33).

### 2.3 Teaching implementation process

#### 2.3.1 Pre-class learning

One week prior to the start of the anatomy course, all students in the three cohorts were provided with learning resources through the online platform. Class A (the control group) accessed traditional MOOC resources, including video lectures and reading materials, which outlined the general objectives of the anatomy course. In parallel, Class B (experimental group one) was provided with observation content to guide their use of the 3D anatomy models. The content focused on observing the positions, morphological structures, and other key aspects of major organs. Students were encouraged to engage in self-guided learning to familiarize themselves with fundamental anatomical knowledge, such as the names, positions, and basic structures of organs. For instance, during the “Axial Skeleton” session, the virtual observation content was provided to Class B students in advance, as outlined in Supplementary Table 1.

Class C (experimental group two) followed a similar process, but with a phased approach. For the first few weeks, Class C students engaged with the same traditional blended learning model used by Class A for the musculoskeletal, visceral, and endocrine systems (phase 1). However, as the course transitioned to studying the vascular system, sensory organs, and nervous system, Class C switched to a blended learning framework that incorporated the 3D anatomy model, similar to Class B’s approach (phase 2). This allowed Class C to experience both traditional learning methods and the integration of 3D technology at different stages of the course.

Ten minutes before the class session, all three groups (Class A, B, and C) participated in a pre-class assessment to evaluate their understanding of the assigned content. This assessment was conducted through an interactive platform known as Rain Classroom, which provided real-time feedback (32). The assessment consisted of ten objective questions designed to evaluate students’ comprehension of the learning materials. Both students and instructors received immediate feedback, helping to identify areas of strength and weakness in the students’ understanding (Table 1).

**TABLE 1:** Stage-specific task completion patterns of Classes A, B, and C throughout the study.

Stages	Class A	Class B	Class C (phase 1)	Class C (phase 2)
Pre-class	Outlining the general objectives of the anatomy course through MOOC resources including video lectures and reading materials			
		Students were provided with observation content to guide their use of the 3D anatomy models		Students were provided with observation content to guide their use of the 3D anatomy models
	Ten minutes before the class session, students participated in a pre-class assessment			
In-class	Students employed 2D illustrations and physical specimens for anatomical study	Students employed 3D models and physical specimens for anatomical study	Students employed 2D illustrations and physical specimens for anatomical study	Students employed 3D models and physical specimens for anatomical study
	At the end of each classroom session, students participated in an in-class assessment			
Post-class	Students were tasked with assignments that primarily involved drawing 2D diagrams	Students utilized the 3D anatomy models for virtual modeling of anatomy structures	Students were tasked with assignments that primarily involved drawing 2D diagrams	Students utilized the 3D anatomy models for virtual modeling of anatomy structures
Mid-term assessment	Midterm exams consist of unit tests covering key systems, administered 1 week after completing each module, with each test comprising 100 subjective questions via “Rain Classroom”			
Final assessment	The final assessment includes a comprehensive exam with both subjective (60 points) and objective (40 points) components, conducted in written format, to conclude the course			

#### 2.3.2 Classroom learning

In-class activities were designed under social learning theory (34), emphasizing observational learning, collaborative modeling, and reciprocal peer interactions to reinforce anatomical knowledge. Students engaged in structured peer discussions, where they observed and internalized problem-solving strategies from peers, while teachers acted as facilitators to demonstrate functional and evolutionary perspectives of human anatomy.

The teaching approach varied across the three cohorts to assess the impact of different interventions: Class A (control) employed 2D illustrations and physical specimens for anatomical study, while Class B (experimental group one) integrated 3D models with physical specimens for multi-modal comparative analysis. Class C (experimental group two) implemented phased interventions: using traditional methods for musculoskeletal, visceral systems, then switching to 3D-physical specimen integration for vascular, sensory, nervous systems (Table 1).

#### 2.3.3 Assessment during classroom learning

Learning outcomes were assessed at the end of each classroom session as in-class test to evaluate material comprehension, using the same format and quantity of assessment as in the pre-class stage (Table 1). Part of the assessment items had been archived in Supplementary Materials 1 (musculoskeletal module) and 2 (neurovascular module).

#### 2.3.4 Post-classroom application

Guided by cognitive load theory (35), which posited that reducing extraneous cognitive load was critical for effective schema construction, this study streamlined post-class assignments by eliminating redundant procedural tasks (e.g., rote memorization exercises).

Class A (the control group) was tasked with assignments that primarily involved traditional 2D diagrams. For instance, students were asked to illustrate and label anatomical structures based on specific clinical scenarios. These assignments required students to demonstrate their understanding of anatomy by depicting the spatial relationships and functions of various body parts, using 2D drawings and textbook references. Class B (experimental group one), in addition to their traditional learning assignments, utilized the 3D anatomy models for virtual modeling of anatomy structures. For example, following the musculoskeletal system module, students utilized 3D anatomical modeling software to identify critical structures and construct biomechanical models, with representative examples shown in Supplementary Figure 1. Subsequently, after completing the venous circulation unit, learners simulated pharmacological pathways—from drug administration via the cephalic vein through hepatic metabolism to final renal excretion—demonstrating pharmacokinetic principles (Supplementary Figure 2). Alternatively, students could visualize the surgical approach in a transjugular intrahepatic portosystemic shunt (TIPS) procedure, employing the 3D model to better understand the anatomy involved in such procedures (Supplementary Figure 3). These assignments aimed to solidify anatomical knowledge and enhance clinical reasoning skills by applying the concepts learned to real-world medical scenarios. Class C (experimental group two) implemented a phased 3D integration protocol: beginning with Class A’s conventional diagrammatic analysis of foundational systems (musculoskeletal, visceral, and endocrine) in phase 1, then adopting Class B’s clinical modeling paradigm for vascular system, sensory organs, and nervous system in phase 2. All assignments were submitted through “Rain Classroom” and evaluated by instructors. Considering varying student proficiency levels in 2D drawing and 3D modeling, these exercises were excluded from formal grading (Table 1).

#### 2.3.5 Midterm and final assessments

Midterm exams consisted of unit tests covering key systems, administered 1 week after completing each module, with each test comprising 100 subjective questions via “Rain Classroom”. The final assessment included a comprehensive exam with both subjective (60 points) and objective (40 points) components, conducted in written format, to conclude the course (Table 1).

### 2.4 Survey instrument development

This study utilized three structured questionnaires to assess distinct pedagogical dimensions. The first questionnaire (Classes B and C) (Tables 2, 3) evaluated participants’ proficiency and experiential engagement with 3D human anatomy models through seven 5-point Likert-scale items with options corresponding to “Strongly agree” (5), “Agree” (4), “Neutral” (3), “Disagree” (2) and “Strongly disagree” (1), focusing on operational competence (e.g., “I can effectively utilize 3D anatomy model applications”) and comparative learning value (e.g., “Integrating 3D models with physical specimens enhances understanding of complex anatomical concepts”), complemented by three open-response questions identifying implementation challenges. The second survey (all cohorts; Tables 4–6) assessed satisfaction with teaching methods (e.g., “This learning approach stimulates your overall satisfaction in anatomy learning”). Since these questions were broader than those in other surveys and harder to assess precisely, we used five 3-point Likert-scale items: “Agree” (3), “Neutral” (2), and “Disagree” (1) (36–38). The third questionnaire (Classes A and B; Table 7) employed a 5-point response format, with options corresponding to “Strongly agree” (5), “Agree” (4), “Neutral” (3), “Disagree” (2) and “Strongly disagree” (1). The items directly measured blended learning impacts on specific competencies (e.g., “Self-motivation, Igniting self-learning motivation, and Summarizing and reflecting on learning methods”). All questionnaires in this study were administered anonymously to ensure participants could provide authentic responses.

**TABLE 2:** Survey instrument items assessing 3D human anatomy model implementation for Class B (*n* = 56) and Class C (*n* = 56).

Survey items	Class B (*n* = 56)	Class C (*n* = 56)	Total (*n* = 112)
**Able to master the use of 3D anatomy model-related apps**			
Strongly agree	43 (76.79%)	39 (69.64%)	82 (73.21%)
Agree	8 (14.29%)	11 (19.64%)	19 (16.96%)
Neutral	4 (7.14%)	5 (8.93%)	9 (8.03%)
Disagree	1 (1.79%)	1 (1.79%)	2 (1.79%)
Strongly disagree	0 (0.00%)	0 (0.00%)	0 (0.00%)
**3D anatomy models are helpful for my anatomy learning**			
Extremely helpful	46 (82.14%)	42 (75.00%)	88 (78.57%)
Helpful	7 (12.50%)	9 (16.07%)	16 (14.28%)
Neutral	2 (3.57%)	5 (8.93%)	7 (6.25%)
Unhelpful	1 (1.79%)	0 (0.00%)	1 (0.89%)
Extremely unhelpful	0 (0.00%)	0 (0.00%)	0 (0.00%)
**By studying with the 3D anatomy model before class, I can grasp general knowledge objectives such as organ names, locations, and morphology**			
Extremely helpful	50 (89.29%)	42 (75.00%)	92 (82.14%)
Helpful	4 (7.14%)	10 (17.86%)	14 (12.50%)
Neutral	2 (3.57%)	4 (7.14%)	6 (5.35%)
Unhelpful	0 (0.00%)	0 (0.00%)	0 (0.00%)
Extremely unhelpful	0 (0.00%)	0 (0.00%)	0 (0.00%)
**Applying the 3D anatomy model and comparing it with actual specimens during class is beneficial for addressing key and challenging issues**			
Extremely helpful	47 (83.93%)	49 (87.50%)	96 (85.71%)
Helpful	7 (12.50%)	5 (8.93%)	12 (10.71%)
Neutral	2 (3.57%)	2 (3.57%)	4 (3.57%)
Unhelpful	0 (0.00%)	0 (0.00%)	0 (0.00%)
Extremely unhelpful	0 (0.00%)	0 (0.00%)	0 (0.00%)
**Applying the 3D anatomy model to virtually model clinical operations after class is beneficial for my understanding of the clinical application of the knowledge learned**			
Extremely helpful	44 (78.57%)	50 (89.29%)	94 (83.92%)
Helpful	9 (16.07%)	4 (7.14%)	13 (1.61%)
Neutral	3 (5.36%)	1 (1.79%)	4 (3.57%)
Unhelpful	0 (0.00%)	1 (1.79%)	1 (0.89%)
Extremely unhelpful	0 (0.00%)	0 (0.00%)	0 (0.00%)
**After class, I will continue to use the 3D anatomy model for learning and expanding my knowledge**			
Extremely helpful	38 (67.86%)	35 (62.50%)	73 (65.18%)
Helpful	16 (28.57%)	16 (28.57%)	32 (28.57%)
Neutral	1 (1.79%)	4 (7.14%)	5 (4.46%)
Unhelpful	0 (0.00%)	1 (1.79%)	1 (0.89%)
Extremely unhelpful	1 (1.79%)	0 (0.00%)	1 (0.89%)
**Using digital virtual models to learn information technology is inspirational for me**			
Strongly agree	33 (58.93%)	40 (71.43%)	73 (65.18%)
Agree	20 (35.71%)	12 (21.43%)	32 (28.57%)
Neutral	2 (3.57%)	3 (5.36%)	5 (4.46%)
Disagree	1 (1.79%)	0 (0.00%)	1 (0.89%)
Strongly disagree	0 (0.00%)	1 (1.79%)	1 (0.89%)
**Which anatomy teaching model is more beneficial for my learning?**			
Traditional classroom teaching	5 (8.93%)	3 (5.36%)	8 (7.14%)
Blended learning	5 (8.93%)	5 (8.93%)	10 (8.93%)
**Traditional classroom teaching with the integration of 3D anatomy models**			
	15 (26.79%)	13 (23.21%)	28 (25.00%)
Blended learning with the integration of 3D anatomy models	28 (50.00%)	31 (55.36%)	59 (52.68%)
Other teaching models	3 (5.36%)	4 (7.14%)	7 (6.25%)
**Advantages of applying digital virtual models in anatomy learning (multiple choices allowed)**			
Spark interest in learning	54 (96.43%)	56 (100.00%)	110 (98.21%)
Enhance self-directed learning abilities	55 (98.21%)	49 (87.50%)	104 (92.85%)
Deepen understanding and memory of knowledge	54 (96.43%)	52 (92.86%)	106 (94.64%)
Enhance the ability to analyze and solve clinical problems	45 (80.36%)	47 (83.93%)	92 (82.14%)
Others	3 (5.36%)	5 (8.93%)	8 (7.14%)
**In what ways does blended learning with digital virtual models promote anatomy learning? (multiple choices allowed)**			
Deepened understanding of abstract concepts	55 (98.21%)	55 (98.21%)	110 (98.21%)
Freedom of learning time and space	55 (98.21%)	56 (100.0%)	111 (99.11%)
Clearer grasp of teaching emphasis and difficulties	54 (96.43%)	51 (91.07%)	105 (93.75%)
Enhanced my application skills in information technology	52 (92.86%)	46 (82.14%)	98 (87.50%)
Did not promote to learning	1 (1.79%)	0 (0.00%)	1 (0.89%)

The dataset presented in this table comprises complete responses from all enrolled participants in Class B (*n* = 56) and Class C (*n* = 56) who completed the 3D model-augmented instructional program.

**TABLE 3:** survey results on the application of 3D human anatomy models for Class B (*n* = 56) and Class C (*n* = 56).

Survey items	Class B (*n* = 56)	Class C (*n* = 56)	*U*-value	*P*-value
Able to master the use of 3D anatomy model-related apps	4.66 ± 0.69	4.57 ± 0.74	1460.0	0.418
3D anatomy models are helpful for my anatomy learning	4.75 ± 0.61	4.66 ± 0.64	1454.5	0.356
By studying with the 3D anatomy model before class, I can grasp general knowledge objectives such as organ names, locations, and morphology	4.86 ± 0.44	4.68 ± 0.61	1346.0	0.052
Applying the 3D anatomy model and comparing it with actual specimens during class is beneficial for addressing key and challenging issues	4.80 ± 0.48	4.84 ± 0.46	1514.0	0.605
Applying the 3D anatomy model to virtually model clinical operations after class is beneficial for my understanding of the clinical application of the knowledge learned	4.73 ± 0.56	4.84 ± 0.53	1404.5	0.136
After class, I will continue to use the 3D anatomy model for learning and expanding my knowledge	4.61 ± 0.71	4.52 ± 0.71	1462.0	0.461
Using digital virtual models to learn information technology is inspirational for me	4.52 ± 0.66	4.61 ± 0.76	1394.0	0.226

This table contains data from Classes B and C participants using 3D model-assisted teaching. The survey showed excellent reliability with Cronbach’s alpha values of 0.967 (Class B) and 0.972 (Class C).

**TABLE 4:** Satisfaction survey results on whether to use 3D human anatomy models in teaching for Class A (*n* = 57) and Class B (*n* = 56).

Survey items	Class A (*n* = 57)	Class B (*n* = 56)	*U*-value	*P*-Value
Stimulating interest in learning	2.63 ± 0.64	2.84 ± 0.42	1364.0	0.061
Achieving learning objectives	2.25 ± 0.79	2.36 ± 0.77	1468.0	0.423
Diverse and interesting teaching activities	2.19 ± 0.74	2.59 ± 0.63*	1123.0	0.003
Amount of extracurricular homework	2.35 ± 0.77	2.55 ± 0.66	1377.0	0.154
Overall satisfaction with the teaching mode	2.51 ± 0.68	2.80 ± 0.44*	1246.0	0.011

This table contains data from control group (Class A) and experimental group (Class B) participants (*N* = 112). The survey demonstrated strong reliability, with Cronbach’s alpha values of 0.964 (Class A) and 0.939 (Class B).

*p < 0.05.

**TABLE 5:** Satisfaction survey results on whether to use 3D human anatomy models in teaching for Class C (*n* = 56).

Survey items	Phase 1 (*n* = 56)	Phase 2 (*n* = 56)	*U*-Value	*P*-value
Stimulating interest in learning	64 ± 0.622	2.91 ± 0.29*	1250.0	0.006
Achieving learning objectives	2.25 ± 0.77	2.43 ± 0.76	1357.0	0.177
Diverse and interesting teaching activities	2.20 ± 0.70	2.70 ± 0.57*	943.0	< 0.001
Amount of extracurricular homework	2.70 ± 0.542	2.52 ± 0.66	1356.0	0.136
Overall satisfaction with the teaching mode	2.36 ± 0.77	2.88 ± 0.33*	1001.0	< 0.001

This table contains data gathered from all Class C participants (*n* = 56) before and after implementing 3D model-assisted teaching. The survey maintained strong reliability throughout both phases, showing Cronbach’s alpha coefficients of 0.952 (phase 1) and 0.912 (phase 2).

*p < 0.05.

**TABLE 6:** Satisfaction comparison of 3D model usage between Class B and Class C (phase 2).

Survey items	Class B (*n* = 56)	Class C phase 2 (*n* = 56)	*U*-Value	*P*-Value
Stimulating interest in learning	2.84 ± 0.42	2.91 ± 0.29	1481.5	0.365
Achieving learning objectives	2.36 ± 0.77	2.43 ± 0.76	1486.0	0.593
Diverse and interesting teaching activities	2.59 ± 0.63	2.70 ± 0.57	1428.5	0.309
Amount of extracurricular homework	2.55 ± 0.66	2.52 ± 0.66	1517.0	0.729
Overall satisfaction with the teaching mode	2.59 ± 0.63	2.88 ± 0.33	1480.5	0.413

This table compares cross-dimensional satisfaction levels between Class B and Class C after 3D model implementation using data from Tables 3, 4. The analysis revealed no significant difference in satisfaction survey scores between Class B and phase 2 of Class C (Cronbach’s alpha = 0.939 for Class B and 0.912 for phase 2 of Class C).

**TABLE 7:** Survey on the impact of different blended teaching models in Class A and B on enhancing college students’ autonomous learning ability.

Survey items	Class A (*n* = 57)	Class B (*n* = 56)	*U*-value	*P*-value
Self-motivation	3.56 ± 1.28	4.09 ± 1.15*	1192.0	0.015
Igniting self-learning motivation	3.75 ± 1.31	4.07 ± 1.14	1378.5	0.187
Summarizing and reflecting on learning methods	3.44 ± 1.17	4.09 ± 1.10*	1054.0	0.001
Self-management of learning	3.75 ± 0.97	3.84 ± 1.04	1513.0	0.605
Self-planning of learning	3.65 ± 1.03	3.79 ± 1.02	1489.0	0.510
Grasping learning patterns	3.70 ± 1.24	4.21 ± 1.06*	1195.5	0.015
Mastering learning strategies	3.86 ± 1.30	4.30 ± 0.95	1311.5	0.077
Flexible learning methods	3.70 ± 1.21	4.34 ± 1.01*	1056.0	0.001
Filtering useful information	3.68 ± 1.24	3.89 ± 1.11	1465.0	0.430
Using online resources for learning	3.81 ± 1.17	4.25 ± 1.08*	1212.0	0.019
Flexibly applying learned knowledge	3.86 ± 1.26	4.36 ± 0.88*	1258.5	0.036
Actively expanding the scope of knowledge	4.07 ± 1.27	4.21 ± 1.11	1528.5	0.669
Identifying problems	3.58 ± 0.92	3.59 ± 0.99	1556.0	0.804
Creating a study plan	3.65 ± 1.03	3.79 ± 1.02	1489.0	0.510
Voluntarily executing the plan	3.42 ± 1.07	3.84 ± 1.09*	1243.0	0.035

This table presents data collected from all participants in Class A and Class B. The questionnaire exhibited excellent reliability, with Cronbach’s alpha values of 0.994 for both Class A and Class B.

*p < 0.05.

### 2.5 Data collection

This study conducted comparative performance analyses between different cohorts. Performance data included scores from pre-class, in-class, mid-term, and final assessments across the three groups. Survey instruments evaluated three dimensions: (a) 3D anatomy model usage in Classes B and C, (b) satisfaction on different blended learning frameworks in all classes, and (c) perceived impacts of 3D anatomy models on self-directed learning capabilities in Classes A and B. Above tables presenting Likert-scale data displayed the scores for different cohorts.

### 2.6 Statistical analysis

Data were compiled in Excel spreadsheets and analyzed using SPSS software (version 29.0). Quantitative data are expressed as means ± standard deviations, while categorical data are presented as frequencies [number of cases (%)]. The normality of the formative assessment results was assessed using the Q-Q plot and the Kolmogorov-Smirnov test (Supplementary Table 2, Supplementary Figures 4, 5). Since the data approximately followed a normal distribution, independent samples *t*-tests were employed to examine the significance of differences in the formative assessment results. The normality of survey responses was assessed using the Kolmogorov-Smirnov test, with p ≥ 0.05 indicating a normal distribution. In this study, none of the survey data met the criteria for normality (Supplementary Tables 3–6). Consequently, the Mann-Whitney U test was used to assess the significance of differences in survey results. A *p*-value of less than 0.05 was considered statistically significant. The reliability of all survey responses was evaluated using Cronbach’s alpha, with a coefficient greater than 0.7 indicating acceptable reliability.

### 2.7 Ethical guidelines

This research strictly adhered to established ethical guidelines governing educational studies. Adherence was maintained to principles of voluntary participation and informed consent, with all subjects receiving detailed notification outlining the research purpose, methodology, and their rights as participants. Specifically, the research protocol incorporated three-layer data anonymization measures: (1) replacement of identifiable information with alphanumeric codes, (2) segregation of demographic data from response records, and (3) secure encryption of digital files. Participants retained unequivocal rights to withdraw consent at any research phase without academic penalty. Class A learners voluntarily abstained from 3D resource utilization during the study period. Classes B and C also voluntarily engaged with the 3D anatomical modeling software for interactive learning modules and model-building tasks in alignment with the experimental protocol. Participants across cohorts maintained oral agreements to avoid curriculum-related discussions. Rigorous monitoring ensured compliance with predetermined learning modalities across cohorts while preserving the integrity of comparative analyses.

## 3 Results

### 3.1 Student formative assessments

#### 3.1.1 Comparison between Class A and B (use of 3D anatomy model)

**FIGURE 1:**
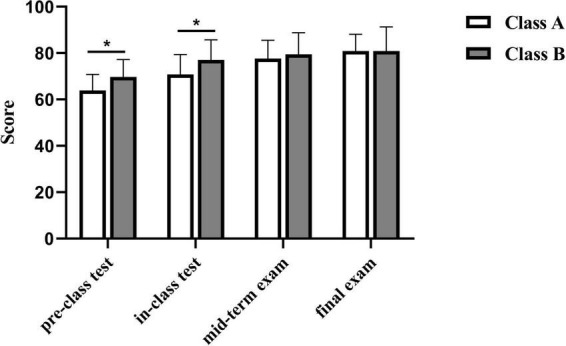
Comparative performance analysis of Class A and B at different time points (*p* < 0.05). *p < 0.05.

In the pre-class test, the average score of Class A was 63.8 ± 6.9 (*n* = 57), while that of Class B was 69.7 ± 7.5 (*n* = 56). Class B demonstrated a statistically significant improvement over Class A (*p* < 0.05). Similarly, in the in-class test, the average scores were 70.8 ± 7.6 (*n* = 57) for Class A and 77.1 ± 8.7 (*n* = 56) for Class B, with a statistically significant difference favoring Class B (*p* < 0.05). These results suggested an immediate benefit of incorporating 3D models into learning. However, in the midterm exam, the average scores were 77.6 ± 8.0 (*n* = 57) for Class A and 79.4 ± 9.4 (*n* = 56) for Class B, while in the final exam, both groups achieved an identical average score of 80.8 (Class A: 80.8 ± 7.3, *n* = 57; Class B: 80.8 ± 10.5, *n* = 56). No significant differences were observed in midterm and final exam scores (*p* > 0.05) (Figure 1).

#### 3.1.2 Analysis for Class C (phase 1 and phase 2)

**FIGURE 2:**
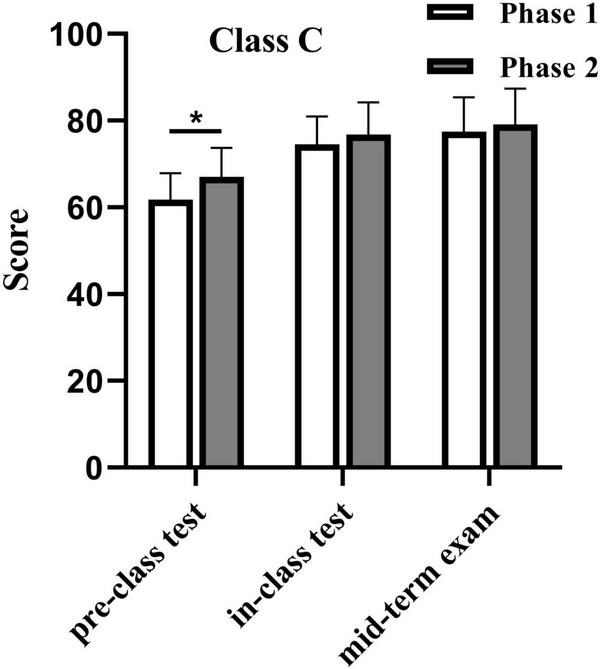
Comparative performance analysis of Class C before and after utilizing 3D human anatomy models (*P* < 0.05). *p < 0.05.

In the pre-class test, before the application of the 3D anatomy model, the average score of phase 1 was 61.8 ± 6.1 (*n* = 56). After implementing the 3D model, the average score in phase 2 increased to 67.0 ± 6.7 (*n* = 56), demonstrating a statistically significant improvement (*p* < 0.05). However, no significant changes were observed in subsequent assessments. In the in-class test, the average score of Class C increased from 74.6 ± 6.4 (*n* = 56) in phase 1 to 76.8 ± 7.5 (*n* = 56) in phase 2. Similarly, in the midterm exam, the average score rose from 77.4 ± 8.0 (*n* = 56) to 79.1 ± 8.2 (*n* = 56) following the intervention, but these differences were not statistically significant (*p* > 0.05) (Figure 2).

### 3.2 Survey results

#### 3.2.1 Students’ usage of 3D anatomy models

A survey of 112 students in Classes B and C demonstrated that over 90% achieved mastery of 3D anatomy modeling applications, with no significant proficiency difference observed between the two classes. Notably, 92.85% found 3D anatomy models beneficial for learning and 93.75% continued using the models for further study and knowledge expansion post-class. The survey demonstrated high reliability, with Cronbach’s alpha values of 0.967 (Class B) and 0.972 (Class C), indicating strong internal consistency (Tables 2, 3).

#### 3.2.2 Satisfaction with blended learning model in Class A and B

The satisfaction survey indicated that students in Class B, who used 3D models, reported higher satisfaction in teaching diversity and overall teaching model satisfaction, compared to Class A (*p* < 0.05) (Table 4). The reliability analysis showed that Cronbach’s alpha was 0.964 for Class A and 0.939 for Class B, confirming acceptable reliability for both groups.

#### 3.2.3 Teaching satisfaction in class C before and after using the 3D anatomy model

Following implementation of the 3D instructional model, Class C showed significant improvement in several learning satisfaction dimensions, notably in three key areas: stimulated learning interest, diversified instructional activities, and enhanced course satisfaction. These improvements demonstrated statistical significance (*p* < 0.05) (Table 5). Reliability analysis revealed satisfactory internal consistency for both administrations, with Cronbach’s alpha coefficients of 0.952 (pre-implementation) and 0.912 (post-implementation).

#### 3.2.4 Satisfaction analysis of 3D model implementation: Class B vs. Class C (phase 2)

Satisfaction analysis demonstrated no statistically significant differences between Class C (phase 2) and Class B regarding the integration of 3D models into the blended learning framework (Table 6).

#### 3.2.5 Impact on autonomous learning skills

Post-implementation comparative analysis identified significant intergroup divergences in specific learning competencies. Class B particularly excelled in self-motivation, summarizing and reflecting on learning methods, grasping learning patterns, flexible learning methods, using online resources for learning, flexibly applying learned knowledge and voluntarily executing the plan, with these differences reaching statistical significance (*p* < 0.05) (Table 7), though not in other measured domains. Both cohorts exhibited exceptional measurement reliability, with Cronbach’s alpha coefficients of 0.994 for Class A and 0.994 for Class B, substantially exceeding recommended threshold of 0.70 for educational research instruments.

## 4 Discussion

Cadaver dissection has been the cornerstone of traditional anatomical education for centuries, offering students a hands-on, tactile approach to learning human anatomy. However, the use of cadavers in educational settings has faced several challenges, including ethical concerns, environmental implications, and the increasing scarcity of cadavers (39). These issues have prompted the exploration of alternative methods to enhance anatomical education. In particular, digital technologies such as including 3D anatomy models have emerged as promising tools to supplement traditional dissection and provide deeper insights into anatomical structures that are often difficult to comprehend using conventional methods (40, 41). Our study aimed to investigate the effectiveness of integrating 3D anatomy models into a blended learning framework for anatomy education. The results showed that the integration of 3D anatomy models enhanced the learning efficiency of student. However, despite these positive effects, there was no significant improvement in final learning outcomes, such as performance on final exams. This finding is consistent with a previous research (42), which suggests that while virtual technologies may enhance engagement, they do not always lead to significant improvements in final assessments or long-term knowledge retention.

Virtual technologies such as VR and 3D anatomy models offer several advantages over traditional dissection methods. VR allows students to explore the human body in a virtual space, offering an immersive experience that enables them to visualize and interact with anatomical structures in a way that traditional dissection does not allow (43, 44). 3D anatomy models provide dynamic, interactive representations of organs, tissues, and systems, allowing students to manipulate and view them from various angles and perspectives. These technologies help to bridge the gap in anatomical education created by the scarcity of cadavers and the ethical concerns associated with their use (45).

Despite the promising potential of virtual technologies, studies have shown that their integration into traditional classroom settings requires careful pedagogical planning and thoughtful incorporation into the curriculum. Some studies suggest that virtual technologies used in isolation, without proper integration into the curriculum, may not significantly enhance learning outcomes (46–48). This is a critical point in understanding why our results, while showing improvements in engagement and immediate learning outcomes, did not lead to substantial improvements in final assessments. These findings suggest that while virtual technologies are beneficial in enhancing student engagement, they must be strategically integrated into existing teaching practices to maximize their potential.

In comparison, other studies have highlighted the profound impact of VR and 3D models on students’ understanding of anatomical structures. These technologies provide students with an interactive and dynamic learning experience that traditional dissection cannot fully replicate (16, 42, 49). For instance, VR allows students to explore anatomical structures from all angles and in great detail, which facilitates a better understanding of complex anatomical relationships. In this context, virtual technologies are particularly valuable in helping students gain insights into structures that are difficult to examine through traditional methods, such as the intricacies of the vascular system or the sensory organs (50, 51).

Our research supports the idea that 3D models can enhance pre-class learning efficiency, as evidenced by improved test scores in the experimental groups. Students in the experimental groups who had access to 3D anatomy models demonstrated a better understanding of anatomical concepts before class, as reflected in their higher pre-class test scores. This improvement in pre-class learning aligns with previous studies that have found 3D models to be effective in enhancing students’ ability to visualize anatomical structures and engage with the material more deeply (52). By providing students with the opportunity to interact with 3D models before attending class, the models helped them become more familiar with the anatomical content and, as a result, improved their preparedness for in-class activities.

However, this improvement in pre-class learning efficiency did not translate into significant improvements in final test scores. This outcome raises an important issue: while virtual technologies like 3D models can enhance students’ engagement and understanding of anatomical structures, this may not always carry over to better performance in summative assessments, especially in complex subjects like anatomy (52). One possible explanation for this lack of improvement in final outcomes could be the complexity of the vascular and nervous systems, which are more intricate and challenging to master. Even with the aid of 3D models, students may find it difficult to grasp the detailed and complex relationships between different anatomical structures, particularly when these structures are interconnected in complex ways.

The complexity of anatomy is a well-established challenge in the field of medical education (53), and while 3D models may assist students in visualizing structures, mastering them remains a difficult task. This issue is compounded by the fact that 3D models alone may not be sufficient to help students fully understand the underlying principles of anatomy. For instance, while 3D models can help students identify and visualize individual anatomical structures, they may not provide the context and understanding necessary to make connections between these structures or understand their functions in a clinical setting (47). As such, the use of 3D models may be most effective when combined with other teaching methods, such as traditional dissection, lectures, and hands-on activities, that allow students to apply their knowledge in real-world situations (54).

Another significant finding from our study is the impact of virtual technologies on student satisfaction. The integration of 3D models into the learning process resulted in higher student satisfaction, which aligns with previous research suggesting that blended learning models incorporating virtual technologies improve student satisfaction (16). Students reported higher levels of engagement and satisfaction with the learning experience, which is an encouraging outcome, as engagement and satisfaction are important predictors of academic success and long-term retention.

However, the increased workload associated with the use of 3D models led to decreased satisfaction in our study. While students appreciated the enhanced learning experience provided by 3D models, the additional homework and study time required to interact with the models placed a strain on their time and energy. This finding highlights an important challenge in the integration of virtual technologies into educational settings: while these technologies can enhance engagement and understanding, they also require careful management to prevent students from feeling overwhelmed by the increased workload. Balancing the introduction of new technologies with realistic expectations for students’ time and energy is critical to maintaining overall satisfaction with the course (55).

This challenge underscores the importance of thoughtfully designing learning activities that integrate virtual technologies in a way that maximizes their benefits without overburdening students. In future studies, it would be important to assess how different levels of integration and workload management can influence student satisfaction and performance (56). It may also be beneficial to explore the potential for adaptive learning systems that tailor the use of virtual technologies to individual students’ needs and progress, thereby optimizing learning outcomes while minimizing unnecessary stress (57).

While the incorporation of 3D models did not result in significant improvements of expanding knowledge or formulating learning plans in the context of autonomous learning capabilities, our study did show that the models enriched students’ understanding of anatomical structures. This aligns with findings from previous research suggesting that while 3D technologies may not immediately enhance autonomous learning skills, they can improve students’ ability to visualize and understand complex structures, which is crucial for the development of deeper, more reflective learning practices (58). The failure to show superior results in autonomous learning compared to traditional methods presents an area for future exploration (59). It may be that while 3D models are effective in enhancing students’ understanding of specific anatomical structures, they do not necessarily foster the development of higher-order learning skills, such as problem-solving, critical thinking, and independent learning. Future studies should explore how virtual models can be used to promote these skills by encouraging students to actively engage with the material, identify gaps in their knowledge, and formulate strategies for further learning.

There are several limitations to this study that should be addressed in future research. This study has several notable limitations: Firstly, the extended temporal scope of the research may have introduced confounding variables due to uncontrolled intergroup interactions and environmental factors that evolved over time. Secondly, our large-cohort design carries inherent contamination risks, as control group exposure to 3D learning resources during the trial period could not be fully excluded, potentially compromising intergroup comparison validity. Thirdly, the inherent complexity differential between anatomical systems studied by Group C creates a fundamental disparity in learning challenge levels. This systemic variation in content difficulty likely influenced academic outcomes independent of instructional methodology, thereby introducing comparative bias in the experimental analysis.

In conclusion, our research indicates that integrating 3D anatomy models into blended learning frameworks can enhance learning efficiency and student satisfaction, though it does not significantly improve final learning outcomes. Exploring how 3D models can be optimized to promote not only short-term learning outcomes but also long-term retention and the development of autonomous learning capabilities is crucial in future. This approach will help maximize the potential of virtual technologies in medical education, offering students new ways to engage with and understand the human body.

## Data Availability

The raw data supporting the conclusions of this article will be made available by the authors, without undue reservation.
