# Supplementary material for: Enhancing anatomy education with virtual reality: integrating three-dimensional models for improved learning efficiency and student satisfaction
Source: Front Med (Lausanne). 2025 Jun 4;12:1555053. doi: 10.3389/fmed.2025.1555053 (PMC12174101; doi:10.3389/fmed.2025.1555053)
Supplement: Supplementary file 1 [file Image_1.pdf]

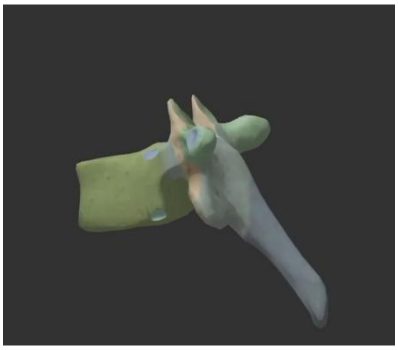

A：Student Works Based on "Visible Body 3D Anatomy"

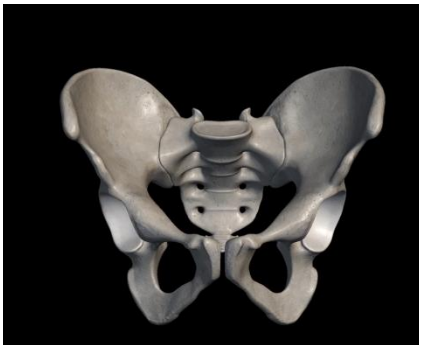

B：Student Works Based on "Anatomy - 3D Atlas"

Supplementary Fig.1 Showcasing selected student works.
