# Supplementary material for: Enhancing anatomy education with virtual reality: integrating three-dimensional models for improved learning efficiency and student satisfaction
Source: Front Med (Lausanne). 2025 Jun 4;12:1555053. doi: 10.3389/fmed.2025.1555053 (PMC12174101; doi:10.3389/fmed.2025.1555053)
Supplement: Supplementary file 2 [file Image_2.pdf]

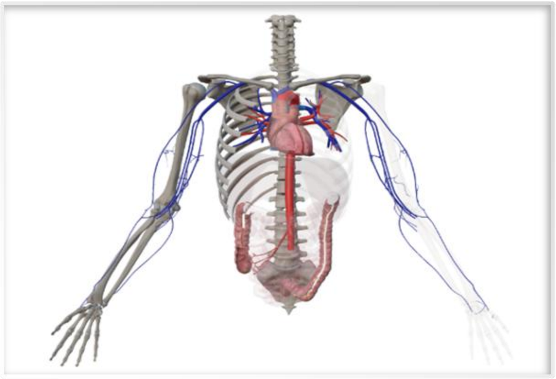

**Supplementary Fig.2** Student post-Class assignment showcase: observation of the Pathway for injecting medication into the appendix through the dorsal venous network of the hand.
