# Supplementary material for: Enhancing anatomy education with virtual reality: integrating three-dimensional models for improved learning efficiency and student satisfaction
Source: Front Med (Lausanne). 2025 Jun 4;12:1555053. doi: 10.3389/fmed.2025.1555053 (PMC12174101; doi:10.3389/fmed.2025.1555053)
Supplement: Supplementary file 3 [file Image_3.pdf]

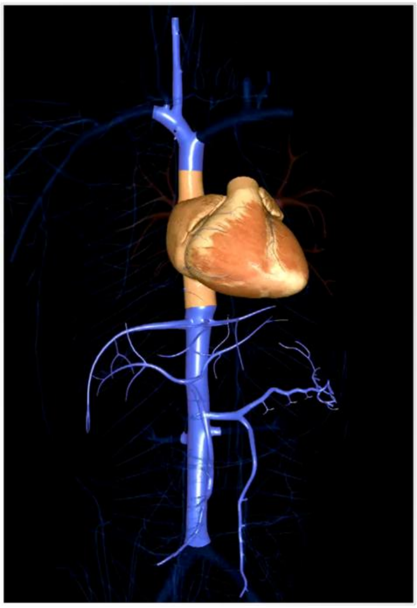

Supplementary Fig.3 Student post-class assignment showcase: observation of the surgical pathway for transjugular intrahepatic portosystemic shunt through the jugular vein.
