# Supplementary material for: Enhancing anatomy education with virtual reality: integrating three-dimensional models for improved learning efficiency and student satisfaction
Source: Front Med (Lausanne). 2025 Jun 4;12:1555053. doi: 10.3389/fmed.2025.1555053 (PMC12174101; doi:10.3389/fmed.2025.1555053)
Supplement: Supplementary file 6 [file Presentation_1.pdf]

Axial Skeleton (Pre-class test)

1. **Composition of the trunk bones ( )**
   A. Vertebrae, ribs, sternum
   B. Vertebrae, ribs
   C. Vertebrae, sacrum
   D. Thoracic vertebrae, ribs
   E. Thoracic vertebrae, sternum, sacrum
   Correct Answer: A

2. **Vertebral foramen ( )**
   A. Formed by adjacent superior and inferior vertebral notches
   B. Formed by the vertebral body and lamina
   C. Formed by the vertebral body and vertebral arch
   D. Formed by the lamina and pedicle of the vertebral arch
   E. Formed by the vertebral body and processes
   Correct Answer: C

3. **Intervertebral foramen ( )**
   A. Formed by the superior and inferior vertebral notches of adjacent vertebrae
   B. Formed by the vertebral body and lamina
   C. Formed by the vertebral body and vertebral arch
   D. Formed by the vertebral body and pedicle
   E. Formed by vertebral articulations
   Correct Answer: A

4. **The atlas corresponds to:**
   A. The 1st cervical vertebra
   B. The 2nd cervical vertebra
   C. The 7th cervical vertebra
   D. The 4th thoracic vertebra
   E. The 5th lumbar vertebra
   Correct Answer: A

5. **Vertebrae with transverse foramina are ( )**
   A. Cervical vertebrae
   B. Thoracic vertebrae
   C. Lumbar vertebrae
   D. Coccygeal vertebrae
   E. Sacrum
   Correct Answer: A

6. **Morphological features of lumbar vertebrae include:**
   A. Heart-shaped vertebral body cross-section
   B. Bifid spinous processes
   C. Broad, plate-like spinous processes
   D. Overlapping spinous processes angled posteroinferiorly
   E. Transverse foramina
   Correct Answer: C

7. **The sternal angle aligns with ( )**
   A. The 1st rib
   B. The 2nd rib
   C. The 3rd rib
   D. The 4th rib
   E. The 5th rib
   Correct Answer: B

8. **A structural feature of ribs is:**
   A. Costal facets
   B. Transverse costal facets
   C. Costal notches
   D. Costal grooves
   E. Xiphocostal angles
   Correct Answer: D

9. **Incorrect description of mandibular morphology:**
   A. Composed of a mandibular body and ramus
   B. The mandibular angle is palpable externally
   C. The external surface of the mandibular ramus contains the mandibular foramen
   D. The mental foramen is located on the anterolateral surface of the mandibular body
   E. The condylar process expands superiorly into the mandibular head
   Correct Answer: C

10. **Correct description of sacral morphology:**
   A. Five pairs of anterior sacral foramina
   B. Formed by fusion of four sacral vertebrae
   C. Articulates with the 4th lumbar vertebra
   D. Contains the spinal cord within the sacral canal
   E. The sacral cornu serves as a landmark for sacral anesthesia
   Correct Answer: E

Axial Skeleton (In-class test)

1. **Incorrect description of trunk bone composition ( )**
   A. Ribs
   B. Vertebrae
   C. Patella
   D. Coccyx
   E. Sternum
   Correct Answer: C

2. **A typical vertebra lacks ( )**
   A. Vertebral body
   B. Vertebral arch
   C. Vertebral foramen
   D. Lateral masses
   E. Spinous process
   Correct Answer: D

3. **The first cervical vertebra ( )**
   A. Also called the axis
   B. Also called the vertebra prominens
   C. Possesses a dens (odontoid process)
   D. Has a long spinous process
   E. Also called the atlas
   Correct Answer: E

4. **Incorrect description of the vertebral arch:**
   A. Located posterior to the vertebral body
   B. Semilunar in shape
   C. Forms the vertebral foramen with the vertebral body
   D. Gives rise to a pair of spinous processes

E. The pedicle is the constricted portion connecting to the vertebral body
Correct Answer: D

5. **Morphological features of thoracic vertebrae include:**
   A. Transverse foramina on transverse processes
   B. Bifid spinous processes
   C. Poorly developed superior/inferior articular processes
   D. Horizontally oriented spinous processes
   E. Costal facets on the posterolateral vertebral bodies
   Correct Answer: E

6. **Incorrect description of the sternum:**
   A. A flat bone
   B. Contributes to the thoracic cage
   C. Composed of a manubrium and body
   D. The sternal angle aligns with the 2nd costal cartilage
   E. Convex anteriorly
   Correct Answer: C

7. **A unique feature of cervical vertebrae is ( )**
   A. Vertebral body
   B. Vertebral arch
   C. Transverse foramina
   D. Transverse processes
   E. Articular processes
   Correct Answer: C

8. **An unpaired cranial bone is ( )**
   A. Parietal bone
   B. Occipital bone
   C. Lacrimal bone
   D. Inferior nasal concha
   E. Palatine bone
   Correct Answer: B

9. **A 40-year-old female undergoes sacral anesthesia for perianal surgery. The landmark for needle placement is ( )**
   A. Sacral promontory
   B. Sacral tuberosity
   C. Sacral cornu
   D. Coccygeal apex
   E. Posterior sacral foramina
   Correct Answer: C

10. **A structure belonging to the facial bones is ( )**
    A. Superior nasal concha
    B. Inferior nasal concha
    C. Frontal bone
    D. Sphenoid bone
    E. Ethmoid bone
    Correct Answer: B
