# Supplementary material for: Enhancing anatomy education with virtual reality: integrating three-dimensional models for improved learning efficiency and student satisfaction
Source: Front Med (Lausanne). 2025 Jun 4;12:1555053. doi: 10.3389/fmed.2025.1555053 (PMC12174101; doi:10.3389/fmed.2025.1555053)
Supplement: Supplementary file 7 [file Presentation_2.pdf]

Heart (Pre-class test)

1. **Correct statement about cardiac surface landmarks:**
   A. Coronary sulcus separates left and right atria
   B. Sulcus terminalis separates atria and ventricles
   C. Interventricular groove overlies the interventricular septum
   D. Apical notch is located on the left side of the cardiac apex
   E. Coronary sulcus lies in the anatomical coronal plane
   Correct Answer: C
2. **All are right atrial structures EXCEPT:**
   A. Fossa ovalis
   B. Coronary sinus orifice
   C. Valve of inferior vena cava
   D. Trabeculae carneae
   E. Pectinate muscles
   Correct Answer: D
3. **Correct description of the right atrium:**
   A. Receives blood only from superior/inferior vena cava
   B. Forms most of the cardiac base
   C. Contains mitral valve at its outlet
   D. Separated from left ventricle by interatrial septum
   E. Medial wall contains fossa ovalis
   Correct Answer: E
4. **Structure belonging to the right ventricle:**
   A. Septomarginal trabecula
   B. Crista terminalis
   C. Tendon of Todaro
   D. Mitral valve
   E. Aortic bulge
   Correct Answer: A
5. **Structure visible in the left atrium:**
   A. Superior/inferior vena cava orifices
   B. Coronary sinus orifice
   C. Fossa ovalis
   D. Crista terminalis
   E. Mitral valve orifice
   Correct Answer: E
6. **Mitral valve complex includes:**
   A. Mitral annulus
   B. Mitral valve leaflets
   C. Chordae tendineae
   D. Papillary muscles
   E. All of the above (A+B+C+D)
   Correct Answer: E

7. **Structure passing through the right fibrous trigone:**
   A. Atrioventricular bundle
   B. Left bundle branch
   C. Right bundle branch
   D. Internodal tracts
   E. Sinoatrial nodal branch
   Correct Answer: A
8. **Incorrect description of the right ventricle:**
   A. Gives rise to pulmonary trunk superiorly
   B. Contributes to the inferior cardiac border
   C. Occupies most of the anterior cardiac surface
   D. Participates in forming the cardiac apex
   E. Superior narrowed portion forms conus arteriosus
   Correct Answer: D
9. **All are right ventricular structures EXCEPT:**
   A. Pectinate muscles
   B. Papillary muscles with chordae
   C. Septal leaflet of tricuspid valve
   D. Supraventricular crest
   E. Tricuspid valve
   Correct Answer: A
10. **Cardiac valve complexes do NOT include:**
   A. Mitral/tricuspid annuli
   B. Valve leaflets
   C. Chordae tendineae
   D. Papillary muscles
   E. Trabeculae carneae
   Correct Answer: E

Heart (In-class test)

1. **Incorrect description of cardiac morphology/location:**
   A. Pyramidal shape
   B. Apex directed left-anterior-inferiorly
   C. Base oriented right-posterior-superiorly
   D. 2/3 located left of midline
   E. Ventricular portion is mobile; atrial portion is fixed
   Correct Answer: E
2. **Correct description of intracardiac structures:**
   A. Coronary sinus opens into left atrium
   B. Right ventricular outlet is aortic orifice
   C. Tricuspid valve connects left atrium and ventricle
   D. Crista terminalis marks left ventricular division
   E. Moderator band present in right ventricle

Correct Answer: E

3. **Valve status during ventricular systole:**
   A. Aortic/pulmonary valves open
   B. Mitral/tricuspid valves open
   C. Aortic valve open, pulmonary valve closed
   D. Mitral closed, tricuspid open
   E. Mitral open, aortic valve closed
   Correct Answer: A
4. **Structures preventing backflow during ventricular diastole:**
   A. Aortic and mitral valves
   B. Pulmonary and tricuspid valves
   C. Aortic and tricuspid valves
   D. Aortic and pulmonary valves
   E. Mitral and tricuspid valves
   Correct Answer: D
5. **Normal cardiac pacemaker:**
   A. Sinoatrial node
   B. Myocardial fibers
   C. Atrioventricular node
   D. Bundle of His
   E. Purkinje fibers
   Correct Answer: A
6. **Landmark dividing right ventricular inflow/outflow tracts:**
   A. Septomarginal trabecula
   B. Anterior mitral leaflet
   C. Supraventricular crest
   D. Trabeculae carneae
   E. Papillary muscles
   Correct Answer: C
7. **Incorrect description of right atrial inlet/outlet:**
   A. Superior vena cava usually lacks valves
   B. Coronary sinus orifice lies deep to crux
   C. Coronary sinus orifice typically has a valve
   D. Outlet contains mitral valve
   E. Valve of inferior vena cava at its anterior margin
   Correct Answer: D
8. **Incorrect statement about interventricular septum:**
   A. Surface landmark: anterior/posterior interventricular grooves
   B. Separates ventricles and right ventricle from left atrium
   C. Mostly muscular, partially membranous
   D. Supplied by anterior/posterior interventricular arteries
   E. Membranous portion common site for defects
   Correct Answer: B

9. **A 78-year-old female with anterior wall STEMI likely has occlusion in:**
   A. Left conus branch
   B. Left posterior ventricular branch
   C. Anterior interventricular artery (LAD)
   D. Left main coronary artery
   E. Right coronary artery
   Correct Answer: C
10. **Landmark dividing right ventricular inflow/outflow tracts:**
   A. Septomarginal trabecula
   B. Anterior mitral leaflet
   C. Supraventricular crest
   D. Trabeculae carneae
   E. Papillary muscles
   Correct Answer: C
