# Supplementary material for: Enhancing anatomy education with virtual reality: integrating three-dimensional models for improved learning efficiency and student satisfaction
Source: Front Med (Lausanne). 2025 Jun 4;12:1555053. doi: 10.3389/fmed.2025.1555053 (PMC12174101; doi:10.3389/fmed.2025.1555053)
Supplement: Supplementary file 8 [file Table_1.docx]

**Supplementary Table 1** Virtual observation structures of the axial bone

| NO. | Modeling Structure | Observation Content |
| --- | --- | --- |
| 1 | General Morphology of Vertebrae | Vertebral Body、Vertebral Arch、Spinous Process、Transverse Process、Superior Articular Process、Inferior Articular Process、Vertebral Foramen、Intervertebral Foramen |
| 2 | Sternum | Manubrium of the Sternum、Body of the Sternum、Xiphoid Process |
| 3 | General Morphology of Ribs | Head of the Rib、Neck of the Rib、Costal Tubercle、Costal Angle |
| 4 | Skull | Cranium、Facial Bones |
| 5 | Mandible | Condylar Process、Ramus of the Mandible、Angle of the Mandible、Masseteric Tuberosity、Pterygoid Fossa |
