# Supplementary material for: Enhancing anatomy education with virtual reality: integrating three-dimensional models for improved learning efficiency and student satisfaction
Source: Front Med (Lausanne). 2025 Jun 4;12:1555053. doi: 10.3389/fmed.2025.1555053 (PMC12174101; doi:10.3389/fmed.2025.1555053)
Supplement: Supplementary file 9 [file Table_2.docx]

**Supplementary** **Table 2** Kolmogorov-Smirnov of the formative assessment results for Class A, B and C.

|  | n P Value |
| --- | --- |
| Class A pre-class | 56 0.011 |
| Class A in-class | 56 0.200* |
| Class A mid-term exam | 56 0.200* |
| Class A final exam | 56 0.008 |
| Class B pre-class | 56 0.164* |
| Class B in-class | 56 0.002 |
| Class B mid-term exam | 56 0.054* |
| Class B final exam | 56 0.002 |
| Class C (before use) pre-class | 56 0.200* |
| Class C (before use) in-class | 56 0.200* |
| Class C (before use) mid-term exam | 56 0.200* |
| Class C (after use) pre-class | 56 0.002 |
| Class C (after use) in-class | 56 0.004 |
| Class C (after use) mid-term exam | 56 0.200* |
