# Supplementary material for: Enhancing anatomy education with virtual reality: integrating three-dimensional models for improved learning efficiency and student satisfaction
Source: Front Med (Lausanne). 2025 Jun 4;12:1555053. doi: 10.3389/fmed.2025.1555053 (PMC12174101; doi:10.3389/fmed.2025.1555053)
Supplement: Supplementary file 10 [file Table_3.docx]

**Supplementary** **Table 3** Kolmogorov-Smirnov of survey results on the application of 3D human anatomy models for Class B (n=56) and Class C (n=56)

| Survey items | | Class B | P value | Class C | P value |
| --- | --- | --- | --- | --- | --- |
| Able to master the use of 3D anatomy model-related apps | n=56 | | <0.001 | n=56 | <0.001 |
| 3D anatomy models are helpful for my anatomy learning | n=56 | | <0.001 | n=56 | <0.001 |
| By studying with the 3D anatomy model before class, I can grasp general knowledge objectives such as organ names, locations, and morphology | n=56 | | <0.001 | n=56 | <0.001 |
| Applying the 3D anatomy model and comparing it with actual specimens during class is beneficial for addressing key and challenging issues | n=56 | | <0.001 | n=56 | <0.001 |
| Applying the 3D anatomy model to virtually model clinical operations after class is beneficial for my understanding of the clinical application of the knowledge learned | n=56 | | <0.001 | n=56 | <0.001 |
| After class, I will continue to use the 3D anatomy model for learning and expanding my knowledge | n=56 | | <0.001 | n=56 | <0.001 |
| Using digital virtual models to learn information technology is inspirational for me | n=56 | | <0.001 | n=56 | <0.001 |
