# Supplementary material for: Enhancing anatomy education with virtual reality: integrating three-dimensional models for improved learning efficiency and student satisfaction
Source: Front Med (Lausanne). 2025 Jun 4;12:1555053. doi: 10.3389/fmed.2025.1555053 (PMC12174101; doi:10.3389/fmed.2025.1555053)
Supplement: Supplementary file 11 [file Table_4.docx]

**Supplementary Table 4** Kolmogorov-Smirnov of satisfaction survey results on whether to use 3D human anatomy models in teaching for Class A (n=57) and Class B (n=56)

| Survey items | Class A | P value | Class B | P value |
| --- | --- | --- | --- | --- |
| Stimulating Interest in Learning | n=57 | <0.001 | n=56 | <0.001 |
| Achieving Learning Objectives | n=57 | <0.001 | n=56 | <0.001 |
| Diverse and Interesting Teaching Activities | n=57 | <0.001 | n=56 | <0.001 |
| Amount of Extracurricular Homework | n=57 | <0.001 | n=56 | <0.001 |
| Overall Satisfaction with the Teaching Mode | n=57 | <0.001 | n=56 | <0.001 |
