# Supplementary material for: Enhancing anatomy education with virtual reality: integrating three-dimensional models for improved learning efficiency and student satisfaction
Source: Front Med (Lausanne). 2025 Jun 4;12:1555053. doi: 10.3389/fmed.2025.1555053 (PMC12174101; doi:10.3389/fmed.2025.1555053)
Supplement: Supplementary file 13 [file Table_6.docx]

**Supplementary Table 6** Kolmogorov-Smirnov of survey on the impact of different blended teaching models in Class A and B on enhancing autonomous learning ability

| Survey items | Class A | P value | Class B | P value |
| --- | --- | --- | --- | --- |
| Self-Motivation | n=57 | <0.001 | n=56 | <0.001 |
| Igniting self-learning motivation | n=57 | <0.001 | n=56 | <0.001 |
| Summarizing and reflecting on learning methods | n=57 | <0.001 | n=56 | <0.001 |
| Self-management of learning | n=57 | <0.001 | n=56 | <0.001 |
| Self-planning of learning | n=57 | <0.001 | n=56 | <0.001 |
| Grasping learning patterns | n=57 | <0.001 | n=56 | <0.001 |
| Mastering learning strategies | n=57 | <0.001 | n=56 | <0.001 |
| Flexible learning methods | n=57 | <0.001 | n=56 | <0.001 |
| Filtering useful information | n=57 | <0.001 | n=56 | <0.001 |
| Using online resources for learning | n=57 | <0.001 | n=56 | <0.001 |
| Flexibly applying learned knowledge | n=57 | <0.001 | n=56 | <0.001 |
| Actively expanding the scope of knowledge | n=57 | <0.001 | n=56 | <0.001 |
| Identifying problems | n=57 | <0.001 | n=56 | <0.001 |
| Creating a study plan | n=57 | <0.001 | n=56 | <0.001 |
| Voluntarily executing the plan | n=57 | <0.001 | n=56 | <0.001 |
